# Lipoxin A_4_-Induced Heme Oxygenase-1 Protects Cardiomyocytes against Hypoxia/Reoxygenation Injury via p38 MAPK Activation and Nrf2/ARE Complex

**DOI:** 10.1371/journal.pone.0067120

**Published:** 2013-06-24

**Authors:** Xiao-Qing Chen, Sheng-Hua Wu, Yu Zhou, Yan-Rong Tang

**Affiliations:** Department of Pediatrics, First Affiliated Hospital of Nanjing Medical University, Nanjing, Jiangsu, People’s Republic of China; University of Cincinnati, United States of America

## Abstract

**Objective:**

To investigate whether lipoxin A_4_ (LXA_4_) increases expression of heme oxygenase-1(HO-1) in cardiomyocytes, whether LXA_4_-induced HO-1 protects cardiomyocytes against hypoxia/reoxygenation (H/R) injury, and what are the mechanisms involved in the LXA_4_-induced HO-1 induction.

**Methods:**

Rat cardiomyocytes were exposed to H/R injury with or without preincubation with LXA_4_ or HO-1 inhibitor ZnPP-IX or various signal molecule inhibitors. Expressions of HO-1 protein and mRNA were analyzed by using Western blot and RT-PCR respectively. Activity of nuclear factor E2-related factor 2 (Nrf2) binding to the HO-1 E1 enhancer was assessed by chromatin immunoprecipitation. Nrf2 binding to the HO-1 antioxidant responsive element (ARE) were measured by using electrophoretic mobility shift assay.

**Results:**

Pretreatment of the cells undergoing H/R lesion with LXA_4_ significantly reduced the lactate dehydrogenase and creatine kinase productions, increased the cell viability, and increased the expressions of HO-1 protein and mRNA and HO-1 promoter activity. HO-1 inhibition abolished the protective role of LXA_4_ on the cells undergoing H/R lesion. LXA_4_ increased p38 mitogen-activated protein kinase (p38 MAPK) activation, nuclear translocation of Nrf2, Nrf2 binding to the HO-1 ARE and E1 enhancer in cardiomyocytes with or without H/R exposure.

**Conclusion:**

The protection role of LXA_4_ against H/R injury of cardiomyocytes is related to upregulation of HO-1, via activation of p38 MAPK pathway and nuclear translocation of Nrf2 and Nrf2 binding to the HO-1 ARE and E1 enhancer, but not via activation of phosphatidyinositol-3-kinase or extracellular signal-regulated kinase pathway.

## Introduction

Myocardial ischemia/reperfusion (I/R) injury is a major challenge during ischemic stroke, circulatory arrest, heart transplantation and cardiothoracic surgery [1], [2]. Previous studies suggested that the myocardial I/R injury was an inflammatory process characterized by recruitment of neutrophils into the ischemic myocardium, excessive production of pro-inflammatory cytokines and toxic compounds when the blood flow in ischemic tissues was restored [1], [2]. The major toxic compounds were reactive oxygen species (ROS) which generated at reperfusion, and activate multiple molecular cascades of inflammation [1], [2], [3]. Heme oxygenase-1 (HO-1) is an important component of the cellular defense enzyme that is induced by and acts against oxidant-induced tissue injury and myocardial I/R injury [3], [4]. The mechanisms by which HO-1 imparts cardioprotection could be via byproducts of the HO-1 enzymatic reaction, bilirubin and carbon monoxide [4]. HO-1 overexpression may also affect the regulation of apoptotic pathway genes like Bcl-2, Bax, and caspases [4]. Cardiac-specific overexpression of human and rat HO-1 in mice protected the heart from I/R injury and prevented the I/R-induced cardiac dysfunction and apoptosis [4]. Similarly, pharmacological upregulation of HO-1 expression also had a significant therapeutic potential in myocardial I/R injury [5], [6], [7].

Lipoxin A_4_ (LXA_4_) is an endogenously produced eicosanoid, inhibits neutrophil recruitment and activation, reduces many cell responses evoked by pathogens and pro-inflammatory cytokines, blocks the generations of pro-inflammatory cytokines and toxic compounds including ROS, promotes resolution of inflammation, and acts as an endogenous “braking signal” in the inflammatory process [8], [9]. LXA_4_ action is mediated by LXA_4_ receptor (ALX) on cellular membrane, which is known as formyl-peptide receptor-like 1 (FPRL1) [10]. Previous studies have shown that activation of ALX by CGEN-855A provided protection against myocardial I/R injury in both murine and rat models (36 and 25% reduction in infarct size, respectively), and the protective effects were accompanied by inhibition of neutrophil recruitment to the injured heart [10]. LXA_4_ mitigated rabbit myocardial I/R injury in which LXA_4_-induced anti-inflammation and suppression of NF-κB activation may play an important role [11]. Besides the anti-inflammatory role of LXA_4_, LXA_4_-evoked expression of HO-1 may be also involved in the LXA_4_-imparted protective effects on myocardial I/R injury. Our speculation is supported by several investigations which demonstrated that LXA_4_ and aspirin-triggered LXA_4_ amplified HO-1 gene expression in human corneal epithelial cells, endothelial cells and lung tissues [12], [13], [14]. Since it remains unclear whether LXA_4_ increases HO-1 expression in cardiomyocytes, and whether LXA_4_-induced HO-1 is involved the LXA_4_-imparted protective role on myocardial I/R injury, the current studies were therefore undertaken to clarify the above questions.

Extensive studies were carried out to explore the signal transduction mechanisms of HO-1 induction. Several reports demonstrated that signaling pathways in HO-1 expression involved in the mitogen-activated protein kinase (MAPK), phosphatidyinositol-3-kinase (PI3-K)/Akt pathways, nuclear factor-E2-related factor 2 (Nrf2), and antioxidant responsive element (ARE) in promoter of HO-1 gene. The transcription factor Nrf2, which interacts with AREs, has recently emerged as a major player in the transcriptional activation of HO-1 [15]. The signaling molecules involved in HO-1 gene induction are activated in an inducer-specific manner and cell-specific manner. For example, tyrosine kinase inhibitors, but not inhibitors of the extracellular signal-regulated kinase (ERK) and p38 MAPK pathways, attenuated induction of the HO-1 by hemin, sodium arsenite, and cadmium chloride in human HeLa cells [16]. Conversely, statins might activate protein kinase G to elicit activations of ERK and p38 MAPK pathways and finally induce HO-1 gene expression [17]. However, nitric oxide stimulated HO-1 gene expression in smooth muscle cells via the activation of the Nrf2/ARE complex, independent of the MAPK or PI3-K/Akt pathways [18]. Up to now, previous studies have not explored the signal transduction involved in LXA_4_-induced HO-1 expressions [12], [13], [14]. LXA_4_ activates p38 MAPK and ERK but not PI3-K in human renal mesangial cells [19]. Our previous data also showed that LXA_4_ slightly increased the phosphorylation of ERK but not PI3-K/Akt in renal mesangial cells and lung fibroblasts [20], [21], and slightly promoted the phosphorylation of ERK and p38 MAPK but not PI3-K in endothelial cells [22]. However, our data did not find LXA_4_ activated ERK and PI3-K/Akt in renal tubular epithelial cells [23]. Pang H et al reported that LXA_4_ blocked lipopolysaccharide-triggered ROS production in human umbilical vein, and the possible mechanism was through Nrf2 pathway [24]. Along this line, we speculated that p38 MAPK, ERK and Nrf2/ARE signal transduction may be involved in the LXA_4_-induced HO-1 expression in cardiomyocytes. Therefore, the present studies were undertaken to determine the above speculations, and rat cardiomyocytes were used and cellular injury was induced by hypoxia/reoxygenation (H/R) injury which was similar to myocardial I/R injury [25].

## Materials and Methods

### 1. Reagents

Dulbecco’s modified Eagle’s medium (DMEM), fetal calf serum (FCS), non-essential amino acids, were purchased from Gibco BRL (Grand Island, NY). Lactate dehydrogenase (LDH) and creatine kinase (CK) assay kits were obtained from Nanjing Jiancheng Bioengineering Institute (Nanjing, China). TRIzol reagents were purchased from Invitrogen (Carlsbad, CA). LXA_4_, LY294002, an inhibitor of the phosphotransferase activity of PI3-K, and SB203580, an inhibitor of p38 MAPK phosphorylation, were obtained from Calbiochem (San Diego, CA). Rabbit anti-rat HO-1 antibodies were obtained of Abcam (Abcam Ltd., Cambridge, UK). Rabbit anti-rat total Akt, serine-phosphorylated Akt (P-Akt), and Nrf2 antibodies were purchased from Santa Cruz Biotechnology (Santa Cruz, CA). Rabbit anti-rat threonine/tyrosine-diphosphorylated ERK1/2 (P-ERK1/2) and total ERK1/2 antibodies were obtained from Bioworld Technology (Minneapolis, MI). Rabbit anti-rat total p38 MAPK and threonine/tyrosine-diphosphorylated p38 MAPK (P-p38 MAPK) antibodies were obtained from Cell Signaling Technologies (Danvers, MA). Enzyme-linked immunosorbent assay kits (ELISA) for HO-1 levels were purchased from Assay Designs (Ann Arbor, MI). HO-1 activity assay kit was obtained from GenMed Scientifics Inc (Genmed Scientifics, Arlington, MA). Lipofectamine 2000 reagents were purchased from Invitrogen Life Tec (Carlsbad, CA). Chromatin immunoprecipitation (ChIP) assay kit was obtained from Upstate Cell Signaling Solutions (Charlottesville, VA). Total and nuclear protein extraction kit and gel shift assay kit were purchased from Active Motif (Carlsbad, CA). Prime Scrpt™ RT reagent kit and SYBR® premix Ex Taq ™ was obtained from Takara Bio Inc (Shiga, Japan). Chemiluminescent horseradish peroxidase substrate was purchased from Millipore Corporation (Billerica, MA). Zinc protoporphyrin-IX (ZnPP-IX), a specific inhibitor of HO-1 activity, PD98059, an inhibitor of ERK1/2 phosphorylation, collagenase type V, taurine, creatine, l-carnitine, laminin, insulin, cadmium chloride (CdCl_2_), rabbit anti-rat cardiac α-sarcomeric actin antibody were purchased from Sigma-Aldrich Chemical Co (St Louis, MO).

### 2. Cell Culture

Primary cultures of cardiomyocytes were prepared from ventricle of a male Wistar rat weighing 115 g as described previously [26]. All experimental procedures were conducted in accordance with the Guide for Care and Use of Laboratory Animals of the US National Institutes of Health (NIH Publication No.85-23,1996). Briefly, myocardium specimen was cut in small pieces and washed, followed by digestion steps with collagenase type V. The preparation was carried out at 37°C, in the presence of 95% O_2_ and 5% CO_2_. Cardiomyocytes were centrifuged to concentrate them in the pellet. The supernatant containing fibroblasts were removed. The cells were suspended in serum-free DMEM containing 5 mM taurine, 5 mM creatine, 5 mM l-carnitine, 26 nM sodium bicarbonate, 2% non-essential amino acids, 1 U/ml penicillin, 0.5 U/ml streptomycin, and 10 nM insulin, and plated in 26-well plates onto 12 mm glass coverslips that had been coated with 20 µg/ml laminin. Only quiescent, rod-shape myocytes were selected for experiments. Cardiomyocyte purity was determined by immunostaining with the antibody against cardiac α-sarcomeric actin and the purity was above 95%. For induction of H/R injury, cells were cultured in D-Hanks solution in a modular incubator chamber (BioSpherix) with 1% O_2_, 5% CO_2_ and 94% N_2_ for 12 h (hypoxia for 12 h), then exposed to atmosphere of 21% O_2_, 5% CO_2_ and 74% N_2_ and cultured with DMEM for 6 h (reoxygenation for 6 h). Before H/R injury, the cells were exposed to LXA_4_ (10 nM) for 12 h with or without coincubation with ZnPP-IX (10 µM) for 12 h, PD98059 (40 µM) for 30 min, LY294002 (10 µM) for 30 min, SB203580 (30 µM) for 30 min or CdCl_2_ (10 µM) for 1 h.

### 3. Cell Viability Assay and LDH and CK Measurement

Cardiomyocyte viability was determined by using methyl-thiozoyl tetrazolium (MTT) assay measuring the mitochondrial dependent reduction of 3-(4,5-dimethythiazol-2-yl)-2,5-diphenyltetrazolium bromide to a dark blue formazan as we previously described [21]. The LDH and CK levels in cellular supernatants were determined by using ELISA kits following the manufacturer’s instructions.

### 4. Real-time Reverse Transcription-PCR Analysis

Cardiomyocytes treated as mentioned above were harvested, and total RNA was isolated by using Trizol reagents. The RNA was reversely transcripted by using PrimeScrpt™ RT reagent kit following the manufacturer's instructions. The sets of HO-1, Nrf2 and β-actin primers were selected by software-aided analysis (Primer Premier 5.0). The following sequence of primers were used for HO-1, 5′-GCTCTATCGTGCTCGCATGA-3′ (sense) and 5′-AATTCCCACTGCCACGGTC-3′ (antisense), amplifying a 319 bp fragment; for Nrf2, 5′-CTCTCTGAACTCCTGGACGG-3′ (sense) and 5′-GGGTCTCCGTAAATGGAAG-3′ (antisense), amplifying a 182 bp fragment. The β-actin was used as internal controls, 5′-CCCTAAGGCCAACCGTGAA-3′ (sense) and 5′-CCGCTCATTGCCGATAGTGA-3′ (antisense), amplifying a 430 bp fragment. Real-time PCR was carried out with StepOne™ real-time PCR system machine (Applied Biosystems, Foster City, CA). Amplification conditions were identical for all reactions: 95°C for 1 min for template denaturation and hot start prior to PCR cycling. A typical cycling protocol consisted of three stages: 15 s at 95°C for denaturation, 30 s at 65°C for annealing, 30 s at 72°C for extension, and an additional 20 s for fluorescent signal acquisition. A total of 40 cycles were performed. The results were analyzed by calculating the Ct values for target genes in the samples.

### 5. Western Blot Analysis

Total and nuclear proteins of the cell lysates were extracted by using protein extraction kits following the manufacturer’s instructions. Protein concentration was estimated by using the Pierce protein assay kit. Protein (30 µg) was loaded for SDS-polyacrylamide gel electrophoresis. Proteins were transfered onto polyvinylidene difluoride membranes with an electroblotting apparatus. The membranes were incubated with antibodies against HO-1 at 1∶2000 dilution, p38 MAPK, P-p38 MAPK, Akt, P-Akt, P-EKR, ERK at 1∶200 dilution and Nrf2 at 1∶100 dilution at 4°C overnight and washed with TBS containing 0.1% Tween-20. The membranes were then incubated with horseradish peroxidase-conjugated secondary antibodies for 1 h. After washing, signals were visualized by chemiluminescent horseradish peroxidase substrate and normalized to tubulin or β-actin or lamin B1.

### 6. Assessment of HO-1 Activity and Levels

HO-1 activity was determined by using HO-1 activity kit according to the manufacturer's instructions. The HO activity values were expressed as picomoles of bilirubin formed per milligram of protein per hour (pmol/mg/h). HO-1 levels (ng/mg) in the cell lysates were measured by using the ELISA kit following the manufacturer’s instructions.

### 7. Immunofluorescence Assay

Cellular HO-1 and Nrf2 localizations were determined by using immunofluorescence assay. The cells were grown on glass coverslips, and then fixed with 4% paraformaldehyde, washed and incubated with blocking buffer for 30 min at room temperature. The cells were then incubated with the antibodies against HO-1 at 1∶200 dilution or Nrf2 at 1∶100 dilution for 1 h at room temperature. Subsequently, the cells were washed and incubated with biotin-conjugated anti-rabbit IgG at 1∶500 dilution, followed by incubation with FITC-conjugated streptavidin for 1 h at room temperature. Coverslips were mounted on slides, and images of labeled cells were visualized by fluorescence microscopy (Axiovert 200 M; Carl Zeiss, Jena, Germany).

### 8. Small Interfering RNA (siRNA)-based Experiment

The cells were seeded into 6-well plates (1×10^5^ cells/well) for 24 h and then transfected with Nrf2 specific siRNA or nonspecific siRNA by using Lipofectamine 2000 reagents. Subsequently, the cells were exposed to H/R injury after incubated with or without pretreatment with LXA_4_ for 12 h. Expressions of HO-1 mRNA and protein in the transfected cells was assessed by real-time RT-PCR and Western blot respectively. The sense strands of Nrf2 siRNA were 5′-CGCUCAGAACUGUAGGAAAAGGAAGAG-3′. Nonspecific siRNA was used to determine the efficiency of Nrf2 specific siRNA transfection.

### 9. HO-1 Promoter Analysis

The sequences for the wild-type E1 enhancer coupled to a minimum HO-1 promoter (E1), and for mutant E1 enhancer (M739) that had its three ARE core sequences mutated, and for dominant-negative Nrf2 mutant (dnNrf2) that had its transactivation domain deleted were synthesized as previously described by Liu XM et al [18]. These promoter/luciferase constructs (1 µg/ml), pCMVβ-galactosidase (1 µg/ml), and a plasmid expressing a dnNrf2 (pEF-F2/Nrf2), and the empty vector were transfected into cardiomyocytes by using lipofectamine 2000 reagents. The cells were cultured for an additional 24 h, and then treated with or without LXA_4_ (10 nM) for 12 h or CdCl_2_ (10 µM) for 1 h. Luciferase activities of reporter enzyme were determined by using a TD-20/20 Turner Designs luminometer and a spectrophotometer.

### 10. Electrophoretic Mobility Shift Assay (EMSA)

Nuclear protein was extracted by using a nuclear protein extraction kit. EMSA was performed by using a gel shift assay kit following the manufacturer’s instructions. Briefly, the nuclear extracts containing total proteins (30 µg) were preincubated with gel shift binding buffer for 10 min, incubated with a biotin-labeled, double-stranded oligonucleotide probe of ARE (3 µg) for 20 min. The oligonucleotide pairs of ARE were 5′-TTTATGCTGTGTCATGGTT-3′ and 5′-AACCATGACACAGCATAAA-3′. Formed nuclear protein- DNA complexes were dissolved in 4% non-denaturing polyacrylamide gels, and electrophoresis was performed under 220 V for 2 h. The gels were dried and active bands were visualized on X-ray films. In antibody supershift assay, Nrf2 antibody (1 µg) was added to the reaction mixture and incubated for 3 h at 4°C prior to the addition of the probe. To assess the specificity of the reaction, competition assays were performed with 100-fold excess of unlabeled consensus oligonucleotide pairs of ARE. The unlabeled probes were added to the binding reaction mixture 10 min before the addition of the labeled probes.

### 11. ChIP Assay

ChIP assays were performed by using the ChIP assay kit following manufacturer’s instructions and as previously described by Liu XM et al [18]. In brief, the cells were lysed in SDS-lysis buffer and then sonicated. The proteins and DNA were cross-linked with formaldehyde. Sheared chromatin was immunocleared with protein agarose-A and a portion of the precleared chromatin was stored and labeled as“input DNA”. The remaining chromatin was immunoprecipated with IgG (control) or Nrf2 antibodies. Protein-DNA complexes were eluted from the antibodies by elution buffer and formaldehyde cross-links were reversed by addition of NaCl and heating at 65°C for 4 h. DNA was purified and PCR was performed by using a primer pair that spanned the mouse HO-1 E1 enhancer. The primers used were: E1 forward, 5′-AAGAGCTCCACCCCCACCCA-3′ and reverse, 5′-GGGCTAGCATGCGAAGTGAG-3′. A 1.5% agarose gel with ethidium bromide was used to separate and examine the PCR products.

### 12. Statistical Analysis

Results are expressed as mean ± SEM. Experimental data were analyzed using one-way ANOVA followed by LSD test by statistical package for social sciences version 14.0 (SPSS, Chicago, IL). Differences were considered to be statistically significant when *P*<0.05.

## Results

### 1. LXA_4_ Alleviated H/R Injury

**Figure 1:**
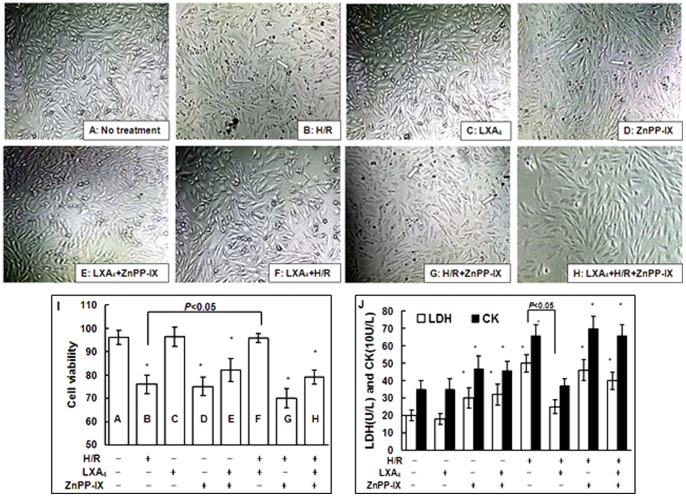
Cellular morphology, viability, LDH and CK release. Figure A to H showed the morphology of rat cardiomyocyte monolayer (×400). Control cells were not treated (A). In B, F, G and H, the cells were exposed to H/R injury for 12 h. In C and D, the cells were treated with LXA_4_ or ZnPP-IX respectively, without H/R injury. The arrows denote the necrotic cells with pyknotic nuclei. In F, cardiomyocytes was pretreated with LXA_4_ for 12 h and then exposed to H/R injury. In E and H, cardiomyocytes were preincubated with LXA_4_ (10 nM) 12 h, and coincubation was continued for 12 h with ZnPP-IX (10 µM) with or without H/R injury respectively. In I, cardiomyocyte viability was as measured by MTT assay. The cells without any treatment (A) served as controls. The letters A to H within the columns represent the same treatment of the cells as shown in Figure A to H. In J, the levels of LDH and CK in the cell supernatants were shown respectively. Results are representative of five independent experiments. Values are means ± SEM, *P<0.05, as compared to the control cells without treatment.

As shown in Figure 1A, normal cardiomyocytes were showed as rod-shaped with full and integral appearance accompanied by good adhesion. H/R injury or treatment with ZnPP-IX alone or combined treatment with H/R injury and ZnPP-IX led to alterations in cell morphology including cell shrinkage, non-fullness and pyknosis/necrosis accompanied by adhesion disability (Fig. 1B, 1D and 1G respectively). However, pretreatment with LXA_4_ significantly protected the cells from the morphological changes induced by H/R injury or ZnPP-IX (Fig. 1F and 1E). Treatment with ZnPP-IX abolished the LXA_4_-imparted protection on H/R injury since the cellular shrinkage and non-fullness appeared (Fig. 1H). Above results are consistent with the cell viability analysis using MTT assay (Fig. 1I). As shown in Figure 1J, the levels of LDH and CK were increased in the cells exposed to H/R injury as compared to the cells without treatment. However, LXA_4_ reduced the H/R injury-enhanced levels of LDH and CK as compared to the cells undergoing H/R injury alone. Additionally, inhibition of HO-1 with ZnPP-IX abolished the cytoprotective property of LXA_4_ on both cell viability (Fig. 1I) and release of LDH and CK (Fig. 1J).

### 2. LXA_4_ Induced HO-1 Expression

**Figure 2:**
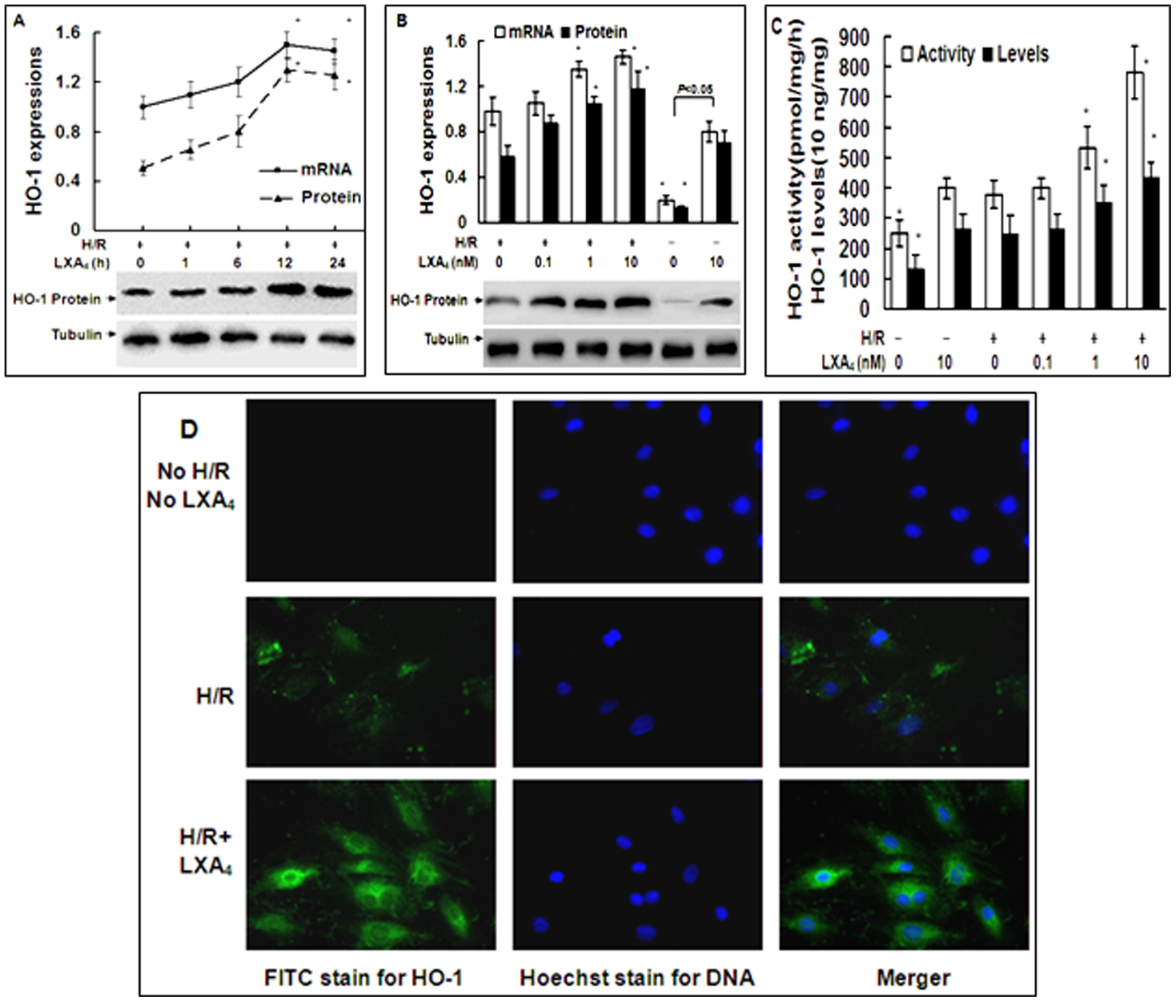
Up-regulation of HO-1 expression induced by LXA_4_ in cardiomyocytes. In A, cardiomyocytes were pretreated with LXA_4_ (10 nM) for 1,6,12 and 24 h before H/R exposure. In B and C, cardiomyocytes were preincubated with LXA_4_ (0, 0.1, 1, 10 nM) for 12 h before H/R exposure. In A and B, the expressions of HO-1 mRNA and protein were measured by using real-time RT-PCR and Western blot respectively. The amount of PCR products was normalized with β-actin to determine the relative expression ratio (log2 fold) for each mRNA. The Western blotting of tubulin protein in lower panel served as a loading control. Protein expression of HO-1 was shown as HO-1/tubulin ratio for each sample. In C, HO-1 activity was expressed as picomoles of bilirubin formed per milligram of protein per hour. The HO-1 levels in cell lysates were assessed by ELISA. In D, the expression and the localization of HO-1 in the cells treated with or without H/R injury and with or without LXA_4_ pretreatment were investigated by using fluorescence microscopy (×400). Values were and mean ± SEM of three independent experiments. *P<0.05, as compared to the cells exposed to H/R injury alone.

HO-1 mRNA and protein expressions were measured in the cells preconditioned with 10 nM LXA_4_ for 1, 6, 12 and 24 h before H/R exposure, and peak expression of HO-1 was induced by LXA_4_ at 12 h (Fig. 2A). Accordingly, expression of HO-1 was detected in cardiomyocytes 12 h after incubation with LXA_4_ at different concentrations (Fig. 2B). As indicated in Figure 2A and 2B, LXA_4_ significantly up-regulated the expressions of HO-1 mRNA and protein in the cells with H/R exposure in a time and concentration-dependent manner. LXA_4_ alone also increased the expressions of HO-1 mRNA and protein in the cells without H/R exposure. Consistently, LXA_4_ upregulated the enzyme activity and levels of HO-1 in a dose-dependent manner in the cells exposed to H/R injury (Fig.2C). LXA_4_ alone also increased the activity and levels of HO-1 in the cells without H/R exposure.

The localization of HO-1 in the rat cardiomyocytes in response to LXA_4_ and H/R injury was also assessed by using fluorescence microscope (Fig. 2D). There was no expression of HO-1 in the cells without H/R injury and LXA_4_ pretreatment. A stronger expression of HO-1 in the cytoplasm of the cells undergoing H/R injury was induced 12 h after the stimulation with LXA_4_ as compared to the cells exposed to H/R lesion alone. Negative controls in each experiment with secondary antibodies alone showed no immunofluorescence (data not shown).

### 3. Role of p38 MAPK, PI3-K/Akt and ERK in HO-1 Expression Induced by LXA_4_

**Figure 3:**
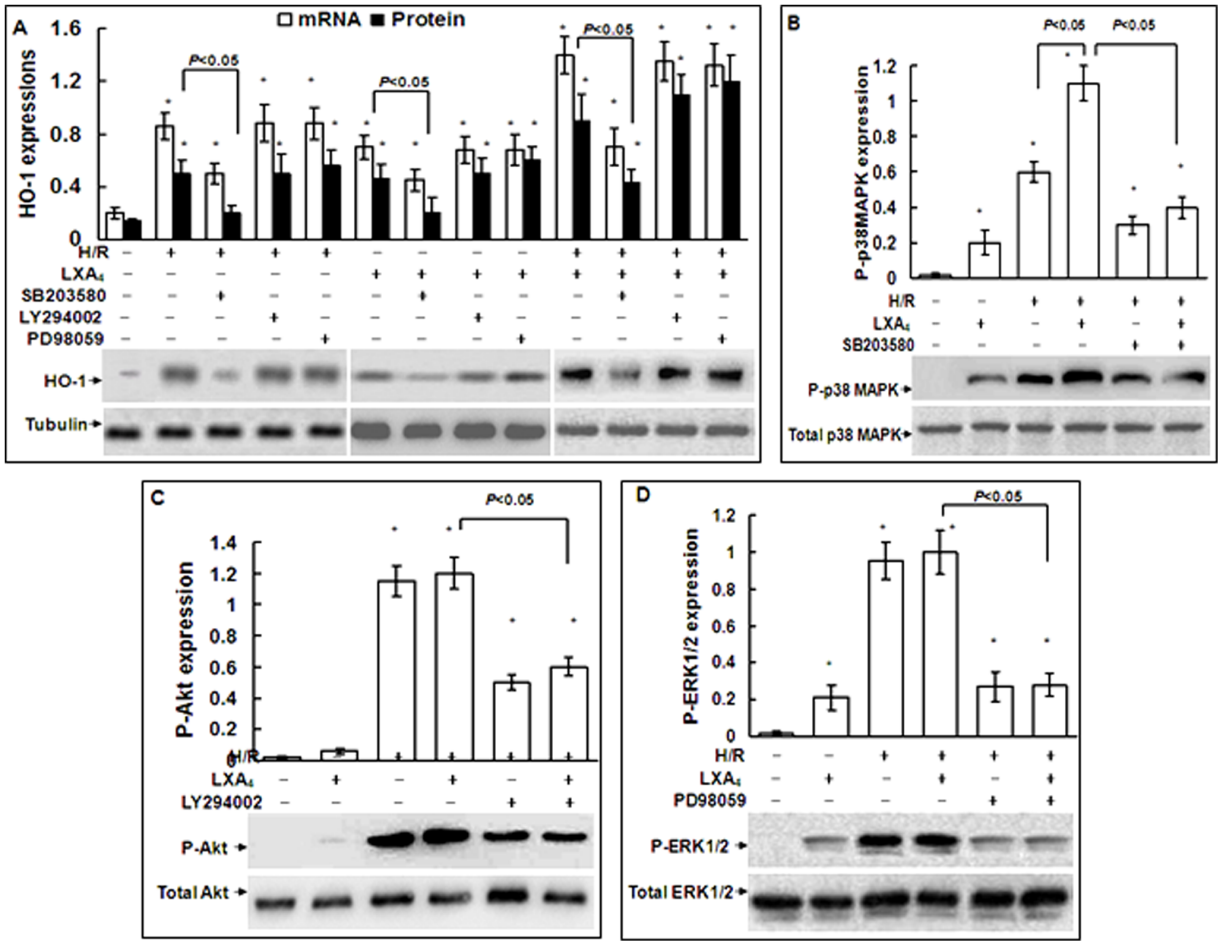
Involvement of p38 MAPK in the expressions of HO-1 induced by LXA_4_. Cardiomyocytes with or without H/R exposure were treated by LXA_4_ for 12 h, SB203580 (30 µM) or LY294002 (10 µM) or PD98059 (40 µM) for 30 min. In A, the expressions of HO-1 mRNA and protein were measured by real-time RT-PCR and Western blotting respectively. The amount of PCR products was normalized with β-actin to determine the relative expression ratio (log2 fold) for each mRNA. Protein expression of HO-1 was shown as HO-1/tubulin ratio for each sample. In B, C and D, total and P-p38 MAPK, total and P-Akt, total and P-ERK1/2 expressions were analyzed by Western blot respectively. Results are representative of three independent experiments. *P<0.05, as compared to the cells without treatment.

The effects of p38 MAPK, PI3-K/Akt and ERK inhibition on expression of HO-1 were shown in Figure 3A. The p38 MAPK pathway inhibitor SB203580 significantly reduced H/R injury-induced, LXA_4_-induced, and LXA_4_ plus HR injury-induced HO-1 expressions, whereas the PI3-K inhibitor LY294002 or ERK inhibitor PD98059 did not. As shown in Figure 3B, 3C and 3D, LXA_4_ alone increased P-p38 MAPK and P-ERK1/2 levels but not P-Akt level in the cells without H/R exposure. H/R injury increased the P-p38 MAPK, P-Akt and P-ERK1/2 levels in the cells without LXA_4_ pretreatment. Furthermore, LXA_4_ further increased H/R injury-induced P-p38 MAPK level but not P-Akt or P-ERK1/2 levels.

### 4. LXA_4_ Induced HO-1 Expression via Nrf2 Translocation

**Figure 4:**
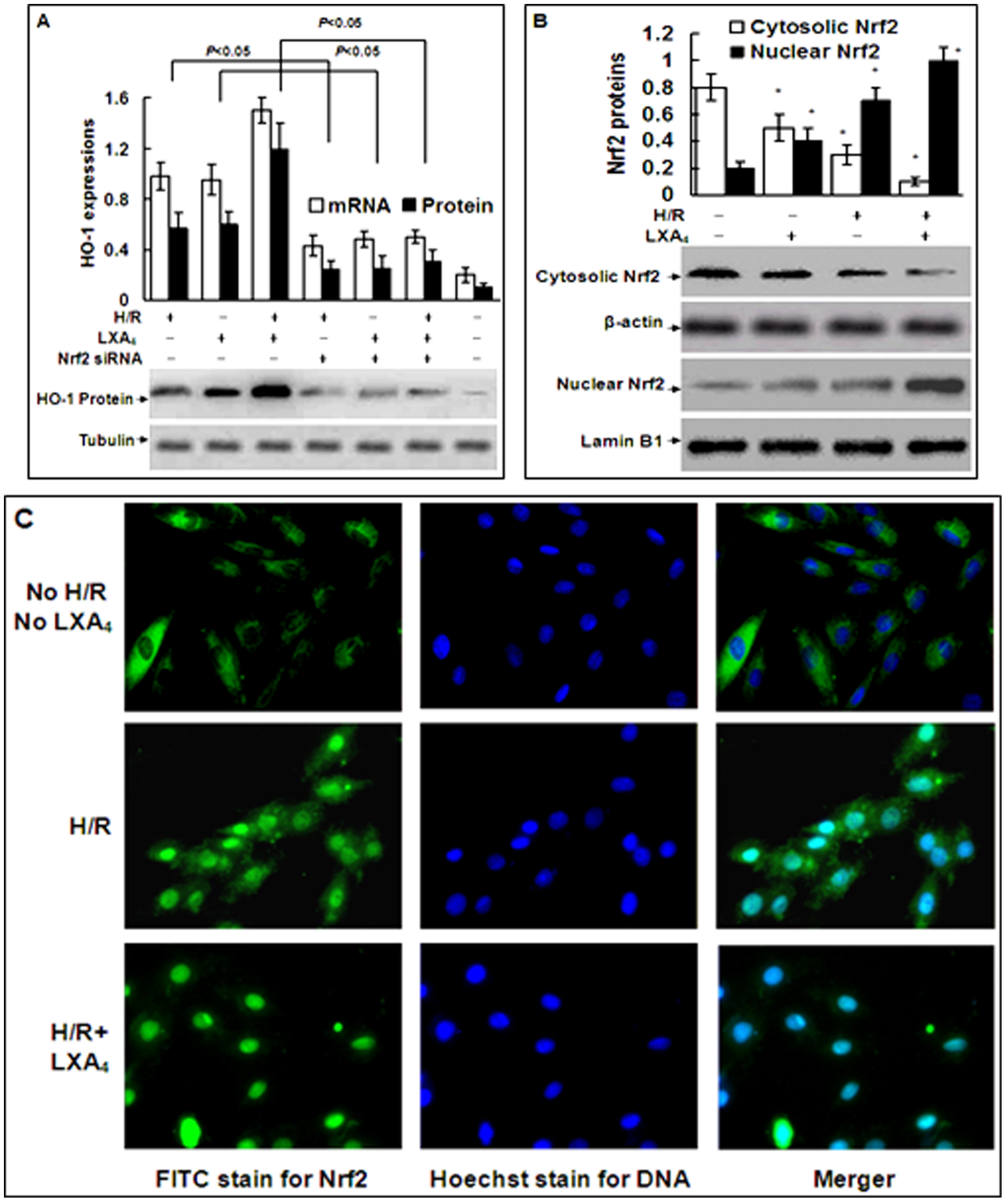
LXA_4_-stimulated HO-1 expression is dependent on Nrf2 translocation. In A, cardiomyocytes were transfected with Nrf2-specific or nonspecific siRNA and then exposed to H/R injury with or without pretreatment with LXA_4_. The effects of Nrf2-siRNA on HO-1 gene and protein expression are shown. The amount of PCR products was normalized with β-actin to determine the relative expression ratio (log2 fold) for each mRNA. Protein expression of HO-1 was shown as HO-1/tubulin ratio for each sample. In B, Nrf2 proteins in cytosolic or nuclear extraction of cardiomyocytes were analyzed by using Western blotting. Cytosolic Nrf2 were shown as Nrf2/β-actin ratio, and nuclear Nrf2 were shown as Nrf2/lamin B1 ratio for each sample. In C, Nrf2 translocation was determined by fluorescence microscope in the cells treated with or without H/R injury and with or without pretreatment with LXA_4_. Results are representative of three independent experiments. In B, *P<0.05, as compared to the cells without treatment.

As shown in Figure 4A, enhanced mRNA and protein expressions of HO-1 induced by LXA_4_, H/R injury and LXA_4_ plus H/R injury were all inhibited by Nrf2-siRNA. As illustrated in Figure 4B, H/R injury induced the Nrf2 translocation from cytoplasm to nuclei, as the Nrf2 protein was decreased in the cytoplasm and increased in the nuclei of the cells after H/R exposure. LXA_4_ alone partially but significantly induced the Nrf2 translocation from cytoplasm to nuclei. LXA_4_ pretreatment further promoted H/R injury-induced Nrf2 translocation. The intracellular translocation of Nrf2 was also detected by fluorescence microscope. As shown in Figure 4C, the Nrf2 protein was only found in the cytoplasm of the cells without H/R injury and LXA_4_ pretreatment. Pretreatment with LXA_4_ further increased H/R injury-induced Nrf2 protein expression in the nuclei, and decreased the expression of Nrf2 in the cytoplasm.

### 5. LXA_4_ Induced HO-1 Gene Transcription via Formation of Nrf2/ARE Complex

**Figure 5:**
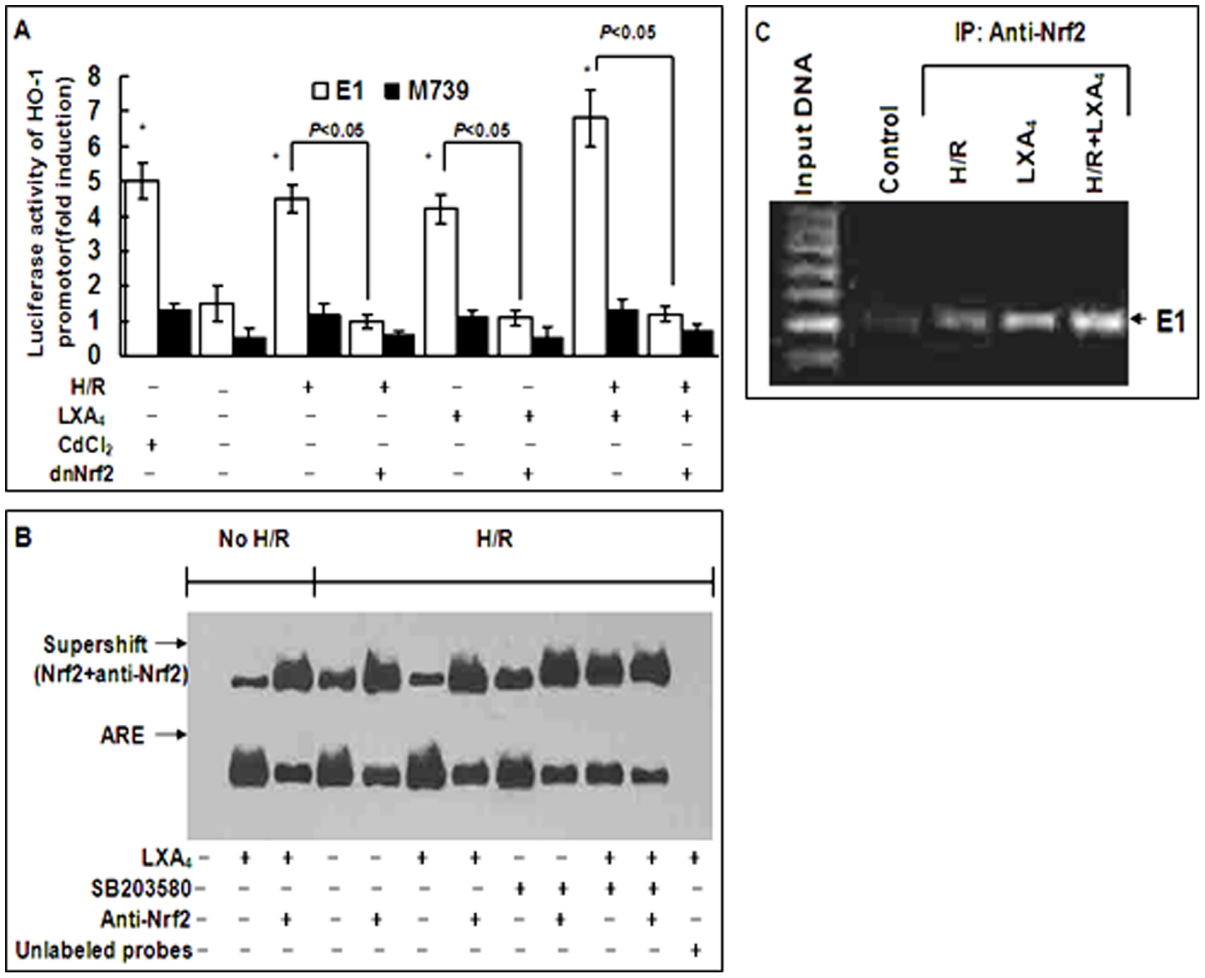
LXA_4_-stimulated HO-1 expression is dependent on formation of Nrf2/ARE complex. In A, cardiomyocytes were transfected with mouse HO-1 promoter construct (E1) or a mutated mouse HO-1 promoter construct (M739) with or without transfection of dominant-negative Nrf2 (dnNrf2), and then treated with H/R injury with or without pretreatment with LXA_4_. Fold induction of luciferase activity of HO-1 promoter was assessed by employing the reporter gene transfection assays. In B, nuclear extracts were prepared from cardiomyocytes, and then subjected to EMSA with biotin-labeled double-stranded oligonucleotide probe of ARE. Supershift assay was performed using an antibody against to Nrf2. In C, ChIP assay determining Nrf2 binding to the HO-1 E1 enhancer following treatment of the cells with H/R injury, LXA_4_ or H/R injury plus LXA_4_. Results are representative of three independent experiments. In A, *P<0.05, as compared to the cells without treatment.

As shown in Figure 5A, CdCl_2_, an established activator of the HO-1 promoter, induced a 5 fold increase in the HO-1 promoter activity. LXA_4_ alone and H/R injury alone could stimulated a 4–5 fold increase in HO-1 promoter activity respectively. Combined treatment with LXA_4_ and H/R injury could stimulate a 6–7 fold increase in HO-1 promoter activity. M739 transfection, mutation of the ARE, abolished the cellular response to LXA_4_, H/R injury and LXA_4_ plus H/R injury. Transfection with dnNrf2 blocked the cellular response to LXA_4_, H/R injury and LXA_4_ plus H/R injury. The formation of Nrf2/ARE complex was also measured by EMSA. As shown in Figure 5B, the DNA binding activity of Nrf2 was enhanced after treatment with LXA_4_, H/R injury and LXA_4_ plus H/R injury. Pretreatment with Nrf2 antibody blocked the DNA binding activity of Nrf2 induced by LXA_4_, H/R injury and LXA_4_ plus H/R injury, and increased the formation of Nrf2-anti-Nrf2 complex. Pretreatment of the cells with SB203580 inhibited the formation of Nrf2/ARE complex induced by LXA_4_, H/R injury and LXA_4_ plus H/R injury. Competition assay carried out with unlabeled oligonucleotide probes indicated the specificity of ARE. The EMSA results are further confirmed by ChIP assays as shown in Figure 5C. ChIP assays with an antibody against Nrf2 revealed the binding of Nrf2 to the E1 enhancer and this binding was substantially enriched after treatment with LXA_4_, H/R injury, and H/R injury plus LXA_4_.

## Discussion

In the present study, we provided the evidence that LXA_4_ protected cardiomyocytes against H/R injury. First, LXA_4_ could reverse morphological changes in the cells treated with H/R exposure (Fig. 1F). Furthermore, the MTT assay also showed that LXA_4_ imparted a cells protection on cellular viability in the cells subjected to H/R injury (Fig. 1I). Finally, LXA_4_ decreased the release of LDH and CK from cardiomyocytes exposed to H/R injury (Fig. 1J).

Previous studies attributed the LXA_4_-induced protection on heart I/R injury to the inhibition of neutrophil recruitment and of pro-inflammatory cytokines production [10], [11]. In the present study, we demonstrated for the first time that LXA_4_-induced HO-1 expression participated in the LXA_4_-imparted protection on heart H/R injury. First, LXA_4_ alone or LXA_4_ plus H/R injury stimulated HO-1 mRNA and protein expressions in cardiomyocytes (Fig. 2A, 2B). HO-1 activity and levels were also upregulated by LXA_4_ in cardiomyocytes with or without H/R injury (Fig. 2C). Furthermore, ZnPP-IX treatment abolished the protection role of LXA_4_ on the cell viability (Fig. 1I) and release of LDH and CK (Fig. 1J) in the cells with H/R exposure. These data suggest that LXA_4_-induced cardioprotection is mediated by HO-1 upregulation in cardiomyocytes exposed to H/R injury. Our results were supported by previous investigations which demonstrated that posttreatment with aspirin-triggered LXA_4_ significantly reduced lipopolysaccharide-induced acute lung injury in mice partially via enhanced formation of HO-1 in the lung tissues [14], and topical treatment with LXA_4_ played anti- inflammatory role during corneal wound healing via restoration of HO-1 expression in 12/15-lipoxygenase (−/−) mice and amplification of HO-1 gene expression in human corneal epithelial cells [13].

Up to present, a plethora of reports have demonstrated that the many signaling pathways were involved in HO-1 transcriptional regulation. The discrepancy results may reflect cell type- and inducer-specific features in the signal regulatory mechanisms [15]–[18]. In the current studies, LXA_4_ alone increased the P-p38 MAPK and P-ERK but not P-Akt levels in the cells without H/R injury (Fig. 3B, 3C and 3D). However, LXA_4_-activated P-ERK may not be involved in the upregulation of HO-1 induced by LXA_4_ since inhibition of P-p38MAPK by SB203580, but not inhibition of P-ERK or PI3-K/Akt, significantly eliminated the upregulation of HO-1 expression induced by LXA_4_ alone, H/R injury, and LXA_4_ plus H/R injury (Fig. 2A). These data indicated that signaling mechanisms involved p38 MAPK phosphorylation is responsible for HO-1 gene activation induced by LXA_4_. Similarly, previous studies have shown that HO-1 expression was induced in cardiomyocytes exposed to oxidative stress or hypoxia via activation of p38 MAPK but not ERK [27], [28]. In cardiac ischemic models, numerous studies demonstrated a crucial role of p38 MAPK [29].

Accumulating data implicated that Nrf2 served as a key regulator of the adaptive response to oxidative stress and of the transcriptional activation of HO-1. Recent studies indicated that Nrf2 also plays an important role in ARE-mediated antioxidant gene expression and the transcriptional activation of HO-1 gene [30], [31]. Pang H et al reported LXA_4_ could prevent the endothelial cells hyperpermeability through activation of Nrf2 in HO-1 induction [24]. However, the role of Nrf2/ARE modulated by LXA_4_ in its cardioprotection remains unknown. The present studies clarified that the translocation of Nrf2 and activation of Nrf2/ARE are essential for LXA_4_-induced HO-1 upregulation in cardiomyocytes. First, the expressions of HO-1 mRNA and protein induced by LXA_4_, H/R injury and LXA_4_ plus H/R injury were inhibited in the Nrf2-siRNA transfection (Fig. 4A). Furthermore, LXA_4_, H/R injury and LXA_4_ plus H/R injury all induced Nrf2 translocation (Fig. 4B). Finally, immune fluorescence experiments demonstrated that LXA_4_ treatment resulted in a marked increase in nuclear Nrf2 staining, confirming that LXA_4_ significantly induced Nrf2 translocation from cytoplasm into nuclei following H/R injury (Fig. 4C).

To determine whether LXA_4_-induced HO-1 expression involves ARE transcriptional activation in HO-1 promoter, cardiomyocytes were transiently transfected with a HO-1 promoter construct E1 and mutant E1 (M739). We found that HO-1 basal transcription was enhanced by LXA_4_ alone and LXA_4_ plus H/R injury, whereas transfection with mutation of the AREs in the HO-1 promoter abrogates the stimulation of HO-1 promoter activity (Fig. 5A). These results indicated that LXA_4_ activates HO-1 transcription via the ARE transcriptional activation. Furthermore, we detected Nrf2/ARE binding in the cells after LXA_4_ pretreatment. EMSA assays revealed that enhanced DNA binding activities of Nrf2 induced by LXA_4_, H/R injury and LXA_4_ plus H/R injury, supershift EMSA reactions using an antibody against Nrf2 retarded the migration of ARE complex, indicating the presence of Nrf2 in the ARE-nuclear protein complex (Fig. 5B). This enhanced Nrf2/ARE complex was dependent on activation of P-p38 MAPK, since p38 MAPK inhibitor SB203580, inhibited the formation of Nrf2/ARE complex induced by LXA_4_, H/R injury and LXA_4_ plus H/R injury (Fig. 5B). ChIP assays provided a direct evidence which demonstrated the Nrf2 binding to the HO-1 E1 enhancer following the treatment of the cells with LXA_4_, H/R injury and LXA_4_ plus H/R injury (Fig. 5C).

In summary, our present experiments indicate that LXA_4_ upregulates the expressions of HO-1 mRNA and protein, which protects cardiomyocytes against H/R injury via activation of p38 MAPK pathway, nuclear translocation of Nrf2 and Nrf2 binding to the HO-1 ARE and E1 enhancer, but not via activation of PI3-K/Akt or ERK pathway. Our results further explained the mechanisms by which LXA_4_ exerts cardioprotection on H/R injury. Recently, growing evidences demonstrated the efficacy of LXA_4_ analogs in treatment of animal arthritis [32], asthma [33], acute lung injury [14], and nephritis [34]. Coupled with previous demonstrations of efficacy of LXA_4_ and activation of LXA_4_ receptor in treatment of myocardial I/R injury [10], [11], our present results represent useful tools for the development of a new drug for treatment of ischemic heart diseases.
